# Galectin-9 gene (LGALS9) polymorphisms are associated with rheumatoid arthritis in Brazilian patients

**DOI:** 10.1371/journal.pone.0223191

**Published:** 2019-10-10

**Authors:** Kamila de Melo Vilar, Michelly Cristiny Pereira, Andrea Tavares Dantas, Moacyr Jesus Barreto de Melo Rêgo, Ivan da Rocha Pitta, Ângela Luzia Branco Pinto Duarte, Maira Galdino da Rocha Pitta

**Affiliations:** 1 Laboratory of Immunomodulation and New Therapeutic Approaches (LINAT), Suely-Galdino Therapeutic Innovation Research Center (NUPIT-SG), Federal University of Pernambuco (UFPE), Recife, Pernambuco, Brazil; 2 Rheumatology Service, Clinical Hospital, UFPE, Recife, Pernambuco, Brazil; 3 Laboratory for Planning and Synthesis of Drugs (LPSF) of the Federal University of Pernambuco (UFPE), Recife, Pernambuco, Brazil; Peter MacCallum Cancer Centre, AUSTRALIA

## Abstract

**Introduction:**

Rheumatoid arthritis (RA) is a chronic autoimmune disease characterized by synovial inflammation and hyperplasia, as well as cartilage and bone destruction. Several proteins are associated with the pathogenesis of the disease. Galectin-9 belongs to the family of lectins that are involved in various biological processes and have anti-inflammatory activity.

**Objective:**

To investigate associations between the SNPs of the GAL-9 gene (LGALS9) and serum levels in rheumatoid arthritis patients. We extracted DNA from 356 subjects, 156 RA patients and 200 healthy controls from northeastern Brazil. Three polymorphisms (rs4795835, rs3763959, and rs4239242) in the LGALS9 gene were selected and genotyped using TaqMan SNP genotyping assay. Serum concentrations of galectin-9 were analyzed by ELISA.

**Results:**

The rs4239242 TT genotype showed a positive association with RA (p = 0.0032, odds ratio = 0.28), and heterozygous TC were prevalent in the control group compared to RA patients (p = 0.0001, odds ratio = 7.99). Galectin-9 serum levels were significantly increased in RA patients compared to the control group (p<0.0001). Patients in remission had high levels of galectin compared to the moderate activity group (p<0.0001). Regarding the Clinical Disease Activity Index (CDAI), patients in remission or low activity presented high levels of galectin when compared to patients in severity (p<0.0001). Patients performing moderate activity had a significant value compared to patients who were in high disease severity (p = 0.0064). Interestingly, the AG genotype (rs3763959) has been associated with a higher presence of bone erosion in RA patients (p = 0.0436). The SNP rs4239242 TT genotype showed a positive association with RA in comparison to the control group. The AG genotype (rs3763959) has been associated with a higher presence of bone erosion in RA patients.

## Introduction

Rheumatoid arthritis (RA) is a chronic autoimmune disease characterized by synovial inflammation and hyperplasia, as well as cartilage and bone destruction, and it may also cause extra-synovial symptoms [1]. The important characteristic of RA is the infiltration of multiple leukocytes into the joints, including B cells, T cells, macrophages, dendritic cells, and neutrophils. Numerous proteins have been shown to play a role in RA pathogenesis, such as autoantibodies used against citrullinated antigens (ACPA). TNFα, IL-1, IL-6, and Th17 cells have recently been identified in the promotion of autoimmune processes, joint destruction, and angiogenesis [2,3]. Its pathogenesis is related to a combination of genetic, environmental, hormonal, smoking, alcohol consumption, and infectious factors, and its progression requires activation of innate immunity [4].

The family of galectins is involved in a range of biological processes. Galectin-9 belongs to the group containing two distinct carbohydrate recognition domains (CRDs) linked by a peptide linker and having anti-inflammatory activity. Galectin-9 is expressed by T cells, macrophages, fibroblasts, and endothelial cells, playing an important role in the regulation of inflammation and immune responses by down-regulating pro-inflammatory T cells [5,6]. An important cell surface receptor for galectin-9 is T cell immunoglobulin and mucin-domain-containing molecule-3 (Tim-3), with an expression on CD4^+^ Th1 cells, CD8^+^ cytotoxic T cells, and CD11b^+^ dendritic cells (DC), but not on Th2 cells or macrophages [7].

Galectin-9 is also related to the regulation of the differentiation of T cell subsets in vitro and in vivo. In cell culture, treatment with galectin-9 induced differentiation of T cells to regulatory T cells (Treg) and suppressed the differentiation of Th17 cells [8]. One study evaluated the expression levels of galectin-9 mRNA in peripheral blood mononuclear cells (PBMCs), where expression was significantly lower in patients suffering from RA with moderate to high disease activity when compared to patients with low disease activity [9].

To date, there are no studies correlating polymorphisms with the susceptibility for rheumatoid arthritis. In this context, our study was the first to analyze whether genetic polymorphisms in the LGALS9 gene were associated with susceptibility for RA development. Based on the broad functions of galectin-9, we hypothesized that functional genetic polymorphism in the gene coding for galectin-9 (LGALS9) would be associated with susceptibility for rheumatoid arthritis.

## Materials and methods

### Patients and controls

RA patient blood samples were acquired from the Rheumatology Outpatient Clinical Hospital Federal University of Pernambuco (UFPE). A total of 156 patients fulfilled four or more American College of Rheumatology (ACR) 1987 classification criteria [10]. Demographic, clinical, and laboratory data were collected from hospital records. Laboratory features of RA patients such as erythrocyte sedimentation rate (ESR) and rheumatoid factor positivity were recorded. Individual disease activity was quantified using the disease activity score (DAS28) [11] and the Clinical Activity Index (CDAI) [12]. The Health Assessment Questionnaire (HAQ) [13] score was also applied to patients to assess functional disability in arthritis. Radiographs of hands were obtained from patients with RA and evaluated for the presence of erosions by an experienced rheumatologist blinded to the clinical data. The control group consisted of 200 matched, unrelated healthy blood donors free of any rheumatologic disease. The clinical data pertaining to patients with RA are presented in Table 1. All subjects gave their written consent to participate. The study was approved by the research ethics committee (CCAE-63648616.0.0000.5208) of the UFPE.

**Table 1:** Demographic and clinical parameters of RA patients and control group.

Nº Controls	200
Female/ male	163/37
Mean Age (min-max)	48.01 (19–82)
**Nº of patients**	156
Female/ male	152/4
Mean age	52.15 (25–77)
**Disease Activity Score 28 joints (%)**	
Clinical remission	72 (46.15)
Moderate disease	47 (30.12)
Severe disease	37 (23.71)
**CDAI (%)**	
Clinical remission	95 (58.97)
Moderate disease	31 (19.87)
Severe disease	30 (19.23)
**HAQ**	
<1	50 (32.05)
>1	106 (67.94)
**Rheumatoid factor (%)**	
Positive	89 (57.05)
Negative	37 (23.71)
**Radiological erosions (%)**	
Present	87 (55.76)
Absent	53 (33.97)
**Erythrocyte sedimentation rate (ESR–mm/h)**	36.54 (0–125)
**Disease modifying antirheumatic drugs (DMARD) **	
Leflunomide	29
Hydroxychloroquine	4
Methotrexate	78
Prednisone	45

### SNP selection and genotyping

Single Nucleotide Polymorphisms (SNPs) for the galectin-9 gene (*LGALS9*) were identified by querying the HapMap. The LGALS9 gene is located on human chromosome 17 (Chr17:25,958,147–25,976,586), with a minor allele frequency (MAF) of at least 0.1% in Caucasians Genomic. The SNPs rs4795835, rs3763959, and rs4239242 were evaluated. Genomic DNA was extracted from the peripheral blood of RA patients and controls using the technical standard phenol-chloroform. Allelic discrimination with TaqMan real-time PCR was used to genotype for polymorphisms in the LGALS9. The PCR products were generated in a 12 μl reaction containing 20 ng DNA, and the cycling parameters were denaturation at 95°C for 10 minutes, followed by 45 cycles of denaturation at 95°C for 15 seconds and the annealing and extension at 60°C for 1 minute in a StepOnePlus Real-Time PCR System according to the conditions recommended by the manufacturer (Applied Biosystems, Foster City, CA, USA).

### Serum Galectin-9 levels determination

Cytokine serum level was determined by Human galectin-9 ELISA Kit (R&D Systems, Ltd., Abingdon, UK) according to the manufacturer’s recommendation. The lower limit of detection for the ELISA galectin-9 kit was 15.6 pg/ml.

### Statistical analysis

All genotype data were checked for derivation from Hardy-Weinberg equilibrium (HWE) using http://www.oege.org/software/hardy-weinberg.html [14]. The differences in allele/genotype frequencies between patients and controls were analyzed by Fisher’s exact test. The association of serum Galectin-9 levels with clinical and laboratory measures of patients with RA were analyzed by univariate comparisons using nonparametric tests (Mann-Whitney tests). A logistic regression analysis was performed to detect potential associations in our case-control sample and associations with clinical parameters. Statistical significance was assumed at p<0.05. Statistical analyses of the data were performed using the GraphPad Prism (version 6.0) statistical program.

## Results

A total of 356 subjects were genotyped, among whom 156 RA patients and 200 controls were age and matched controls. The mean age for RA patients and controls was 52.15 ± 12.10 years and 48.01 ± 12.40 years, respectively. Information on the main clinical characteristics of the RA patients is summarized in Table 1.

**Fig 1:**
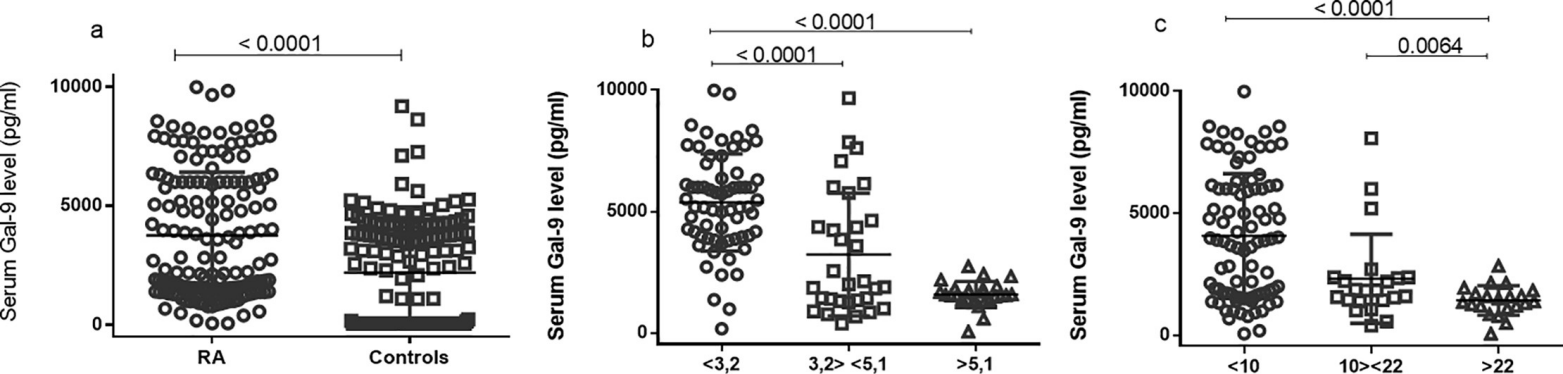
Association of serum Galectin-9 levels in patients with rheumatoid arthritis. (A) serum levels of galectin-9 in patients with RA and control group. (B) association of serum levels of galectin-9 with disease activity score (DAS28). (C) association of serum levels of galectin-9 with Clinical Disease Activity Index (CDAI).

We examined serum galectin-9 levels of RA patients and the control group as shown in Fig 1. Galectin-9 serum levels were significantly increased in RA patients compared to the control group (median 27.82 pg/ml; p<0.0001) (Fig 1A). Patients in remission (DAS28<3.2) had high levels of galectin compared to the moderate activity group (DAS28 3.2> <5.1) (p <0.0001). We evaluated the group in remission in relation to the group with high disease activity(DAS28>5.1) (p <0.0001) (Fig 1B). This finding may be related to mechanisms not yet known or to the use of drugs that modify the course of the disease, as shown in Table 1.

The genotype frequencies of rs4239242, rs3763959, and rs4795835 were distributed according to the Hardy–Weinberg equilibrium in normal controls (all p>0.05). The genotypes and alleles’ frequencies in RA patients and control individuals are shown in Table 2. The rs4239242 TT genotype showed a positive association with RA (15.59% in RA patients and 5.20% in controls, p = 0.0032, odds ratio = 0.28; 95%IC = 0.12–0.64). Heterozygous TC were prevalent in the control group (66.14%) compared to RA patients (19.26%; p = 0.0001, odds ratio = 7.99; 95%IC = 4.59–13.91). Comparison of the homozygous CC versus TC genotypes showed association between patients and controls (odds ratio = 3.36; p = 0.0054; 95%IC = 3.36–7.64). The rs3763959 AA genotype showed a negative association with RA (6.67% in RA patients and 15.10% in controls, p = 0.040, odds ratio = 2.49; 95%IC = 1.05–5.90). Homozygous GG were prevalent in RA patients (55.23%) compared to the control group (34.89%; p = 0.0009, odds ratio = 0.43; 95%IC = 0.26–0.70). Comparison of the homozygous GG versus AG genotypes showed association between patients and controls (odds ratio = 0.40; p = 0.040; 95%IC = 0.16–0.95). On the other hand, the allele and genotype frequencies of rs4795835 showed no association with RA (Table 2).

**Table 2:** Distribution of the LGALS9 alleles and genotypes in patients with RA and healthy individuals.

*SNP genotype*	*RA*		*Control*		*p-value*	*RR*	OR (95%IC)
***rs4795835***	***(N = 154)***	***%***	***(N = 195)***	***%***			
CC	103	66.88	109	55.89	0.063	0.83	0.65 (0.42–1.01)
CT	44	28.57	80	41.03	***0*.*025***	1.25	1.69 (1.08–2.64)
TT	7	4.54	6	3.07	0.415	0.76	0.59 (0.20–1.75)
CT and TT	51	33.11	86	44.10	0.063	1.20	1.52 (0.98–2.34)
C- allele	250	81.16	298	76.41	0.233	0.90	0.79 (0.54–1.13)
T-allele	58	18.83	92	23.58	0.233	1.10	1.26 (0.87–1.81)
***rs3763959***	**(N = 105)**	***%***	**(N = 192)**				
AA	7	6.67	29	15.10	***0*.*040***	1.29	2.49 (1.05–5.90)
AG	40	38.09	96	50	0.052	1.18	1.62 (1.00–2.63)
GG	58	55.23	67	34.89	***0*.*0009***	0.73	0.43 (0.26–0.70)
AG and GG	98	93.33	163	84.89	***0*.*040***	0.77	0.40 (0.16–0.95)
A-allele	54	25.71	154	40.10	***0*.*0001***	1.83	4.20 (2.90–6.09)
G-allele	156	74.28	230	59.89	***0*.*0004***	1.55	1.93 (1.33–2.80)
***rs4239242***	**(N = 109)**	***%***	**(N = 192)**				
TT	17	15.59	10	5.20	***0*.*0032***	0.54	0.28 (0.12–0.64)
TC	21	19.26	127	66.14	***0*.*0001***	2.03	7.99 (4.59–13.91)
CC	71	65.13	55	28.64	***0*.*0001***	0.55	0.22 (0.13–0.36)
TC and CC	92	84.40	182	94.79	***0*.*0054***	1.79	3.36 (1.48–7.64)
T-allele	55	25.22	147	38.28	***0*.*0019***	1.21	1.77 (1.23–2.55)
C-allele	163	74.77	237	61.71	***0*.*0019***	0.82	0.56 (0.39–0.80)

Regarding the Clinical Disease Activity Index (CDAI), patients in remission or low activity (CDA<10) presented high levels of galectin when compared to patients in severity (CDA>22) (p<0.0001). We also evaluated patients with CDAI>10<22 (moderate) and CDAI>22, where patients in moderate activity had a significant value compared to patients who were in high disease severity (p = 0.0064) (Fig 1C). The Health Assessment Questionnaire (HAQ) score to assess functional disability in rheumatoid arthritis and rheumatoid factor was also evaluated, but no significant differences were found based on galectin-9 levels.

**Fig 2:**
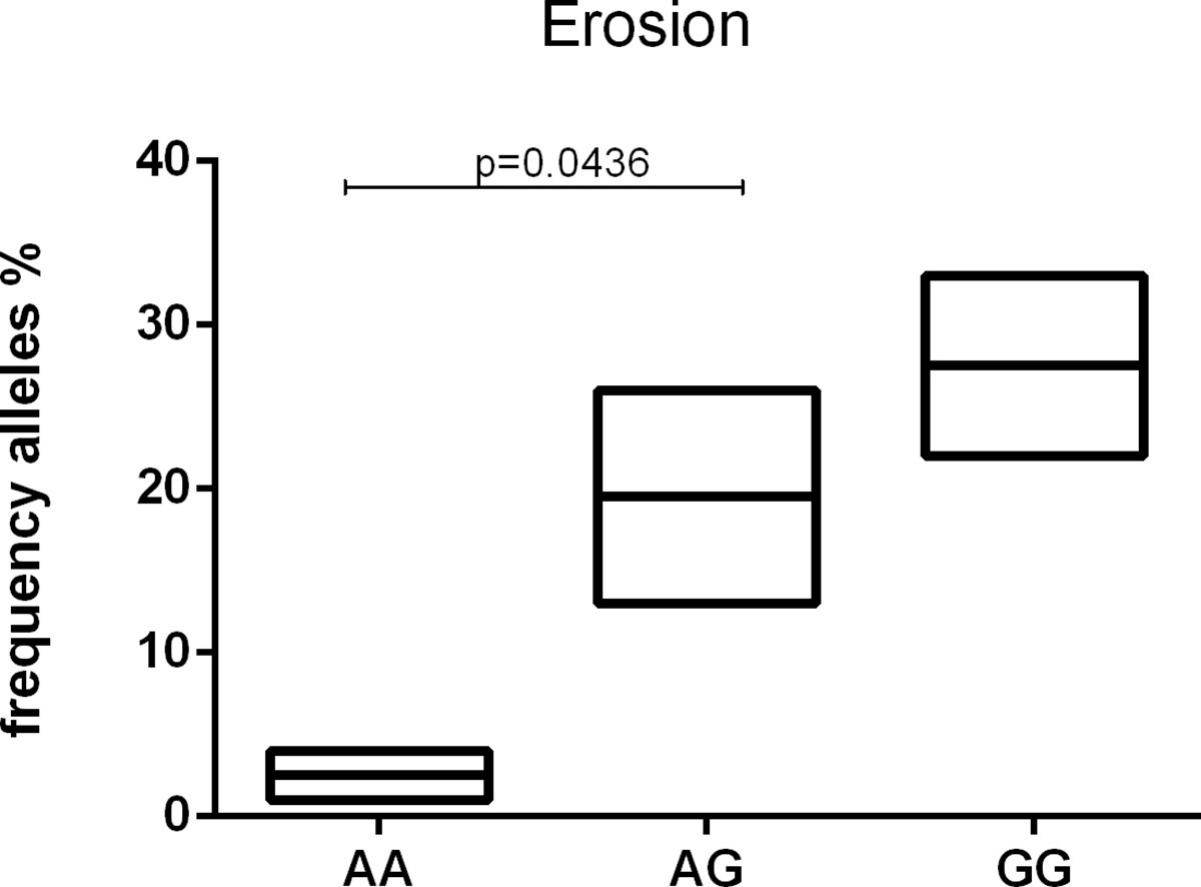
Erosion stratified by genotypes of LGALS9 gene polymorphisms (rs3763959) in RA patients.

To address the functional significance of the selected polymorphisms in the galectin-9 gene, we examined serum levels of galectin-9 with each genotype of RA patients, however, genotypic and allelic frequencies were not associated with serum levels. There were no statistical associations between galectin-9 polymorphisms with DAS28, CDAI, HAQ, ERS, or rheumatoid factor positivity. Interestingly, the AG genotype (rs3763959) has been associated with a higher presence of bone erosions in RA patients (p = 0.0436) (Fig 2).

## Discussion

Many studies have evaluated the role of polymorphisms in galectin genes in rheumatoid arthritis [15,16,17,18]. However, to date there is no study on polymorphisms in the galectin-9 gene in RA. Therefore, this study was carried out to investigate the association of LGALS9 gene polymorphisms in Brazilian patients with rheumatoid arthritis. We did not find associations between the polymorphisms selected and the serum levels of galectin.

Our results show the first positive association of the rs4239242 TT genotype with RA. While genotype AA rs3763959 showed a negative association with RA, genotype frequencies of rs4795835 showed no association with RA. Rosen et al. [19] reported that LGALS9 polymorphisms are associated with the risk of developing alcoholic liver disease (ALD), where an association between the T rs4239242 allele and the protection against ALD and the presence of the C allele were associated with a 70% difference in the risk of developing ALD. This result differs from that found where the TT genotype showed a positive association with RA (p = 0.0032).

The rs3751093 was evaluated, and the proportion of G allele carriers was significantly higher among subjects who were at risk of developing alcoholic liver injury compared to those who were more likely to have the A allele bring on ALD. For different diseases, the polymorphism can generate diverse effects in the individual [19]. The AG genotype (rs3763959) has been associated with a higher presence of bone erosion in RA patients (Fig 2). Moriyama et al. [20] suggest that galectin-9 is indeed present in the bone marrow cavity in vivo. The Tim-3 surface receptor is crucial for the exogenous function of galectin-9, where this complex is related to the induction of apoptosis of CD4+ T-helper 1 (Th1), Th17, CD8+ cells, and cytotoxic T cells. Therefore, galactin-9 is an important molecule in the regulation of the immune system due to induction of apoptosis in Th1 and Th17 cells because the activation of stem cells controls the inflammatory process [21]. In order to clarify the Tim-3/gal-9 complex, a large amount of galectin-9 was administered to rats with arthritis, and bone destruction was consequently observed. A recombinant galectin-9 was administered, respectively, and it was found that recombinant galectin-9 lightly suppressed joint inflammation, however, the suppression did not reach significance according to clinical scores. Thus, the bone destruction that accompanies chronic inflammatory disease permeated by osteoclasts can be attenuated by the presence of the potential control system Tim-3/galectin-9, especially at the sites of destruction in RA, indicating that the presence of this complex Tim-3/galectin-9 can lead to increased inflammation and inflammatory bone destruction [20].

There have been no reports regarding an association between rheumatoid arthritis and LGALS9 polymorphism, although galectin-9 has been shown to play an important role in RA pathophysiology [22,23]. Patients with RA are associated with an inflammation in chronic synovium that leads to hyperplasia and activation of a set of fibroblasts similar to macrophages that express degradative enzymes, causing the destruction of joint structures. Several cell types are recruited into the inflammatory environment that develops within the synovium, where the fibroblasts, macrophages, and proinflammatory B and T cells contribute significantly to the pathology of the disease [24,25].

Most polymorphisms evaluated (genotypes and polymorphic alleles) had a protective effect. Pearson et al. [21] evaluated the difference in abundant galectin-9 expression in synovial fibroblasts compared to dermal fibroblasts. Thus, endogenous galectin-9 protects against apoptosis and increases the viability of synovial fibroblasts [9]. Other studies have found that galectin-9 is being used in patients with RA and may be beneficial in collagen-induced arthritis of mice when administered exogenously. In RA patients, a decrease in galectin-9-Tim-3 complex signalling was observed. Tim-3 expression levels in CD4^+^ T cells from RA patients were lower compared to those from healthy controls, resulting in a decrease in galectin-9 mediated apoptosis of CD4^+^ T cells [26]. Thus, it is suggested that this galectin has both pathogenic and pro-inflammatory roles.

One of the limitations of our study was that the vast majority of patients were in clinical remission, and perhaps because of this the frequency of protection alleles was higher and might not have shown the actual function of the polymorphism. Another limitation was the evaluation of the functional significance of the polymorphisms selected in the galectin-9 gene. When we examined serum levels of galectin-9 with each genotype of RA patients, however, genotypic and allelic frequencies were not associated with serum levels. Taking into account that AR is a microenvironment-dependent disease, polymorphisms may provide some function with galectin in the synovial fluid rather than at the systemic level. In addition, this polymorphism may be influenced by other proteins related to the synthesis, excretion, or degradation of galectin-9. Nevertheless, our study is the first to investigate the role of LGALS9 polymorphism in RA patients, contributing to the knowledge of the genetic background of RA.

## Conclusion

Our results suggest for the first time that genetic alterations in the LGALS9 gene are associated with rheumatoid arthritis in the Brazilian population. The SNP rs4239242 TT genotype showed a positive association with RA in comparison with the control group. As the AG genotype (rs3763959) has been associated with a higher presence of bone erosion in RA patients, these findings suggest that polymorphisms in LGALS9 may be an interesting aspect of rheumatoid arthritis for further exploration.

## Supporting information

S1 File Supporting Information files. (XLSX)
